# Retraction Note: Umbilical cord tissue-derived mesenchymal stem cells induce apoptosis in PC-3 prostate cancer cells through activation of JNK and downregulation of PI3K/AKT signaling

**DOI:** 10.1186/s13287-018-1113-9

**Published:** 2018-12-27

**Authors:** Ihn Han, Miyong Yun, Eun-Ok Kim, Bonglee Kim, Min-Hyung Jung, Sung-Hoon Kim

**Affiliations:** 1 Cancer Preventive Material Development Research Center, College of Oriental Medicine, Kyung Hee University, 1 Hoegi-dong, Dongdaemun-gu, Seoul, 130-701 Republic of Korea; 2 School of Medicine, Kyung Hee University, Seoul, 130-701 Republic of Korea

**Retraction Note: Stem Cell Res Ther**

**https://doi.org/10.1186/scrt443**

This article [1] has been retracted by the authors because, contrary to the statement in the article, ethical approval was not obtained to conduct this study. This has been confirmed by Kyung Hee University Research and Ethics Committee. All authors agree to this retraction.

## References

[1] Han I, Yun M, Kim E-O, Kim B, Jung M-H, Kim S-H (2014). Umbilical cord tissue-derived mesenchymal stem cells induce apoptosis in PC-3 prostate cancer cells through activation of JNK and downregulation of PI3K/AKT signaling. Stem Cell Res Ther..

